# Retraction: A facile sonochemical protocol for synthesis of 3-amino- and 4-amino-1,2,4-triazole derived Schiff bases as potential antibacterial agents

**DOI:** 10.1371/journal.pone.0238168

**Published:** 2020-08-20

**Authors:** The PLOS ONE Editors

After this article [1] was published, concerns were raised about similarities between results reported in this article and in previous works.

Specifically:

- Concerns were raised that Figs 1 and 2 report data that were generated by other researchers and reported previously in Figures 10 and 12 of [2]. The journal office did not receive documentary evidence to confirm that the authors had permissions from the data owners to include these results in the *PLOS ONE* article. According to the first author, the spectra obtained by the authors were of poor resolution but were similar to those in [2], so the representative spectra from [2] were included in [1]. The names of the researchers who generated the data were included in Figs 1 and 2, but neither the Results section text nor the figure legends indicate that the data in Figs 1 and 2 were obtained by researchers outside the author group. The first author apologized for not citing the prior work as the source of these results.
- Similarities were noted between chemical analysis data reported for 26 compounds in the Methods section (#6, 10–23, 26–36) and data reported previously in [3–11]. In response to this issue, the first author commented that the *PLOS ONE* article [1] presented a new methodology (sonochemical synthesis) to synthesize compounds which have been reported previously, and compared compounds synthesized using this method to compounds generated using previously reported methods. According to the first author, the Methods section ought to have cited [3] for the conventional method of synthesizing triazole based Schiff bases, and similar spectroscopic data were obtained in [1] and in [3–11] because the same compounds were analyzed in these articles. Underlying data were provided to support some of the results for which similarities were raised, but the first author stated that the full dataset is not accessible at this time due to the current coronavirus-related lockdown situation in Pakistan. Without the full original dataset to support the results in question the *PLOS ONE* Editors cannot fully clarify this issue.

In light of the above concerns, the *PLOS ONE* Editors retract this article.

AS, SLR, MK, and MaM agreed with retraction. MR and MS did not comment as to their position on the retraction decision. MHUR, NS, AI, IU, MM, and KKM either could not be reached or did not respond directly.

Questions have also been raised about the authorship and contributions to the article. This matter will be deferred to the relevant institutions as per guidance of the Committee on Publication Ethics.
